# To be or not to be a facilitator of reflective learning for medical students? a case study of medical teachers’ perceptions of introducing a reflective writing exercise to an undergraduate curriculum

**DOI:** 10.1186/s12909-016-0624-2

**Published:** 2016-04-04

**Authors:** Kanokporn Sukhato, Sutida Sumrithe, Chathaya Wongrathanandha, Saipin Hathirat, Wajana Leelapattana, Alan Dellow

**Affiliations:** Department of Family Medicine, Faculty of Medicine, Ramathibodi Hospital, Mahidol University, Bangkok, Thailand; Medical Education Unit, Faculty of Medicine, Ramathibodi Hospital, Mahidol University, Bangkok, Thailand

## Abstract

**Background:**

Introducing reflective writing to a medical curriculum requires the acceptance and participation of teachers. The purpose of this study was to explore medical teachers’ views on the benefits of introducing a reflective writing exercise into an undergraduate medical curriculum, including their levels of satisfaction and their concerns. We also investigated effects on the teachers’ personal and professional development arising from their roles as novice facilitators.

**Methods:**

A qualitative approach was employed using semi-structured interviews. During an attachment to Primary Care Medicine course, fourth-year medical students (*n* = 180) in the Faculty of Medicine, Ramathibodi Hospital, Bangkok, Thailand were assigned to write a reflective essay titled, “A Significant Event in My First Clinical Year”. After reading the essays and facilitating between one to three small group discussions based on these, each of the 18 teachers enrolled in our study completed an in-depth face to face interview. Transcripts of these were studied, using thematic content analysis to identify emerging themes.

**Results:**

The novice facilitators felt that facilitated reflection was both valuable and appropriate for students. They also perceived that it had a positive impact on their own personal and professional lives. In the early phase of implementing this activity, teachers expressed concerns about 1) their ability and confidence as facilitators in small group discussion 2) their ability to deal with emotions raised within their groups 3) the effectiveness of the activity 4) poor presentation and possible fabrication of student work.

**Conclusions:**

Most teachers regarded this activity as being beneficial to them, to student learning, and to the curriculum. Their insights, including concerns about the level of skill needed for facilitation, provide valuable material for planning a comprehensive faculty development programme.

## Background

During the past few years the development of professional identity has been a major focus of medical education, emphasising the multifaceted and individualised process through which students develop into physicians [1, 2]. Although many factors influence the process of socialisation through which professional identity is formed, reflection in experiential learning plays an important part in shaping a physician’s professional identity [3].

One approach to developing reflective skills in medical students is to use written reflection about personal significant events followed by small group discussion, in which teachers encourage students to focus on learning experiences drawn from their writing [4, 5]. In medical education, medical teachers play an important role in facilitating student reflection. They can help students to convey their experiences more explicitly, identify feelings or perceptions that underlie their behaviour, formulate future plans, interact openly with other students in the group and develop generic skills, such as active listening [6–9].

Several studies report attempts to incorporate reflection into a medical curriculum [5, 10–12], the impact of reflection on medical students’ perceptions [13–17] and the educational features that promote the development of reflective practice [4, 18]. In contrast, given the importance of the role, studies describing facilitators’ perceptions and experiences are relatively scarce. Two studies from nursing and dental education reported that teachers as facilitators recognized the educational benefits of the reflective process for their students [19] but another, from the field of medical education, reported that tutors and educators had an incomplete understanding of reflection [20]. There is no evidence in medical education regarding the features of reflective learning that attract medical teachers to participate in this approach or the perceived personal benefits to them as lifelong learners.

Addressing reflective learning within a medical curriculum has a substantial impact on teachers both in terms of time commitment and the requirement to master important new skills [9]. The purposes of this study are to explore medical teachers’ views and experiences about 1) levels of satisfactions with the reflective learning process 2) the overall benefits of this activity 3) their expectations and concerns regarding the implementation of reflective writing in the undergraduate medical curriculum 4) the effects of facilitation on the personal and professional development of the teachers. Results from this will help in the formulation of a conceptual framework for faculty development, including a strategy for instructor engagement and participation.

## Methods

This qualitative study was undertaken using in-depth, semi-structured interviews. Participants in the reflective activity were medical teachers from the Family Medicine Department, Ramathibodi Hospital, Bangkok, Thailand. Each was interviewed by two experienced Family Medicine teachers trained to conduct in-depth interviewing. Each interview lasted approximately 30–60 min.

### Study context

The undergraduate medical curriculum in Thailand is a traditional 6-year course, the first 3 years being devoted to basic sciences and the last three to clinical sciences. At the Faculty of Medicine, Ramathibodi Hospital, individual clinical clerkships had become academically isolated and experiential student learning was largely haphazard and opportunistic. Therefore, to meet new educational aims, the Primary Care Medicine curriculum was modernized in 2010 by introducing a 20-week integrated course, including remodeled clerkships in Family Medicine, Internal Medicine and Surgery. The new collaborative curriculum provides greater opportunities for inter-departmental integration and encourages the systematic use of reflective learning. This also helps students to develop the concept of integrated primary care working and to recognise the responsibilities and roles of other professionals.

Our new curriculum emphasises the importance of the systematic use of reflective learning, students being helped to use reflection to analyse their experiences, define underlying concepts and consider alternative ways of approaching problems. Supervised reflection is a compulsory component of teaching and includes evaluation of each student’s ability to use this as part of the learning process. At the beginning of the Primary Care course, a feature of the new curriculum was to assign each fourth-year medical student to write an essay entitled, “A Significant Event in My First Clinical Year”. Students were advised that the essay should consist of at least two pages and should be referenced where appropriate. Explicit objectives and instructions about key aspects of reflection were given, together with an example of a reflective essay. After submitting their work, students were allocated to groups of three or four and participated in a 3-h group discussion facilitated by a teacher from the Family Medicine Department. Facilitators were given a remit to highlight the affective domains of the students’ experiences and to encourage critical thinking in a collaborative learning environment*.* Our study was devised to explore the impact of this new exercise upon the facilitators.

### Staff development

The concept of reflective learning was introduced to Family Medicine teachers in 2006 with a 3-h seminar on its meaning and benefits. Following this, reflection was incorporated into routine teaching such as brief reflective talks after out-patient sessions and reflecting upon learning experiences through role play. However, the use of reflection was not compulsory and learning outcomes were not evaluated. The reflective writing activity was therefore developed to make the use of reflection more systematic. In the year before the launch of this activity, an orientation exercise was conducted to determine objectives and to develop instructions and conceptual descriptions of the learning process. Additionally, prior to the reflective writing exercise, teachers also received a written framework for reading narrative essays and giving feedback [21, 22]. This preparatory work was undertaken by Family Medicine teachers with a medical education background and an interest in reflective practice and mentoring.

### Data collection

Following completion of the small group discussions, data was collected from the teachers through in-depth interviews. The interview guide was comprised of two sections; 1) demographic characteristics, including previous experience of using reflection in daily life and teaching 2) questions covering feelings towards the implementation of reflection in the course, situations that made respondents feel impressed or discouraged, and perceived benefits of this activity to students and themselves. Data from each interview was tape-recorded and transcribed verbatim, in the Thai language, by a research assistant. All data was kept confidential and was accessible only to the principal investigator (KS). All participants signed informed consent forms. The study protocol was approved by the Institutional Review Board of the Faculty of Medicine, Ramathibodi Hospital.

### Data analysis

In our study, three investigators (KS, SS and WL) independently used coding and analysis to develop themes in areas that repeatedly emerged from the data. An analytical process involving a number of interconnected stages was then used to classify and organize themes and emergent categories. The process was repeated several times to develop interpretation of the interviews and to determine relationships within the results.

## Results

Every Family Medicine teacher (*n* = 18) participated in our in-depth interviews. Most of the instructors were young women (Table 1) and two thirds had experience of using reflection, either in their personal or professional lives. The range of teaching experience was between 1 and 30 years. Each teacher had facilitated one to three small group discussions and had read four to twelve reflective essays. Each of the student reflective essays (*n* = 154) was about non-biomedical issues taken from clerkship experiences.

**Table 1:** Demographic characteristics of the 18 medical teachers in the Family Medicine Department, Faculty of Medicine, Ramathibodi Hospital

Characteristics	Number
Gender	
Male	5
Female	13
Age	
31–40	15
41–50	1
51–60	2
Years of teaching	
< 5 years	9
5–10 years	7
> 10 years	2
Previous experience of reflective writing (personal or professional)	
Yes	12
No	6
Previous experience of using reflection in teaching	
Yes	11
No	7

Analyses of the teachers’ interviews revealed four major thematic categories and these are used as our main headings. Themes from within these categories are presented with illustrative quotations from the teachers, translated from the Thai language.

### Perspectives towards using reflective writing

In response to the question, “Please rate your satisfaction with using reflective writing in the new curriculum,” each teacher gave a satisfaction score greater than 7 using a 10-point-Likert scale. Teachers reported that the activity enhanced personal and professional fulfillment and renewed their enthusiasm. Involvement in this activity made their routine teaching more stimulating.

#### Feeling content and fulfilled

Teachers perceived this activity as an attractive teaching and learning experience for them.

“… *Despite all my frustrations and disappointments with the medical profession nowadays, joining this activity encourages and inspires me a lot to stay (in this profession). While reading their stories I was able to sense determined motivation among medical students, showing their intention to become good doctors…” (T25)*

Several reported that the energy and innocence of their young students helped them to revisit their own experiences as medical students and to re-experience the idealism of becoming a good doctor. They were reminded of their own inherent kindness and compassion.

*“We have become dehumanised after years of practice. The overload and demanding routine activities make us become less sensitive to everything. Reading these stories has helped me to be more aware of the patient’s distress and able to see the relationship between professionalism and compassion. This activity reminds me who I was.” (T20)*

*“After the students told me how badly they felt when their patients died, I realized how much I have lost the sensitive feelings I used to have when I was a young medical student.” (T23)*

#### Appreciating students’ thoughtfulness and humanistic thinking

Many teachers saw their students’ empathy, courage and thoughtfulness for the first time, changing their attitudes towards these young students.

*“I admire these students. They are enthusiastic about understanding their patient’s feelings. They can capture these impressionistic and interesting moments and learn from them. This is beyond my expectation that students usually learn only by rote learning.” (T20)*

*“One student brought pillows and a blanket from his home to a patient. This is something I think I would never do. This is the way he showed how he cared for the feelings of his patient. His courage****s****urprised me.” (T4)*

#### Fostering reflection in teachers about their roles and identities

Teachers should help students to develop other competencies besides those required for biomedicine**.** They reflected on how their thoughts about their roles and identities as medical teachers had been shaped after being facilitators. Most agreed that this new activity was beneficial to students and that they could play a role in fostering the students’ professional and personal development in areas such as communication, ethics, and professionalism.

*“The activity led the students to a heightened awareness of ethics, professionalism, communication and interpersonal issues. It allowed them to have the time and opportunity to learn about these aspects with a deeper degree of understanding. We should assess the students’ competence in areas in which they may have deficiencies. We can also pick up student problems in some cases. So we can use this information and modify it to make appropriate guidance to fit with areas each student needs, such as ethical aspects. This is an important role for teachers.” (T24)*

Some of the students’ stories gave a picture of the performance they expected from their teachers. This made teachers aware that their students are watching them at all times and that they should be more careful about what they say and do.

*“Some of the sentences in their essays are very striking. This makes me aware how the students are influenced by our behaviour and manner. I also recognise that we should be approachable teachers, but students should not cross the line. There are things we can learn indirectly from what other teachers do, which rarely happens in daily routine.” (T25)*

Facilitation is an important educational technique that teachers should master and the teachers appreciated the facilitation skills they had developed through this activity*.* Some reported feeling challenged by the need to help students to develop deeper learning and critical thinking from their experiences.

*“This is a new teaching activity that stimulates faculty physicians to be facilitators. When I first used this method I felt very enthusiastic. I have to read and analyse essays in order to help students both to get the most out of it and to gain a deeper learning experience.” (T26)*

### Concerns about being a facilitator of reflective learning

In response to the question, “Why did you not give the full score when you rated your satisfaction with using reflective writing in the new curriculum,” teachers reported common concerns that could be divided into four categories.

#### Insecurity about being a facilitator

A common concern among Thai novice facilitators was a feeling of insecurity about the teaching competencies necessary for the different content and approach required by facilitation. Most importantly*,* a few teachers remarked on the difficulties of assessing their own effectiveness as facilitators.

“*I’m not sure I can do it right for the students to get the maximum benefit from this activity. I don’t have enough confidence to teach because sometimes I get frustrated that I can’t find the solutions for some of their problems.” (T25)*

*“It is very challenging to facilitate non-biomedical content. I’m not sure how good I was as a facilitator. We don’t know how much students get from the activity. We don’t know if our facilitation skill is effective enough to help the student to learn critically from his experience.” (T23)*

Facilitating reflective small group discussion is seen as being more complex than other types of small group activity in medical education due to the nature of the shared content. Concerns about managing group dynamics within the sessions were identified by many teachers. They also recognized the need to manage conflict between students, to encourage quiet students to talk and to handle unexpected situations.

*“I felt challenged by some students who did not explore deeper into themselves, no matter how I raised my questions. This also affected the group dynamic in general.” (T23)*

*“Conflict between students in the group is my concern. They did not listen to their friend’s story but (instead) gave a bad and critical reaction when listening to the story.” (T15)*

#### Dealing with emotion

Teachers expressed feelings such as sadness, sympathy and empathy towards their students’ experiences. Dealing with their emotions during the sessions seemed to be a major concern for some, with some preferring to hide their feelings.

*“I am afraid of an unexpected situation. I thought it was OK to talk about this particular issue. But when I talked about it with medical students they unexpectedly burst into tears. I didn’t know how to handle this.” (T9)*

*“I nearly cried after I had listened to the story. As the facilitator, I was struggling to control my feelings, from getting more emotionally involved in the story.” (T6)*

*“I sometimes felt depressed or burdened by an overwhelming story. It is very difficult to be neutral as a group facilitator.” (T17)*

#### Uncertainty about the effectiveness of the new learning strategy

As with any new learning method in medical education, teachers were confused about the intended outcomes of the new strategy.

*“I’m not sure the students can practice in the way they told me in the class. The reason for this is that we don’t know what will happen to them in the future and the social trend inclines towards materialism.” (T2)*

*“I expected the activity to change students into better students or at least to change their attitudes but I don’t think this activity could have an impact on students’ attitudes. No matters how their attitudes are at the baseline, I think their attitudes are not changed. We always have a mix of good students in every class. Even without this session, a good student will continue to be so and to think as a good student.” (T10)*

#### Annoyance with poor presentation and fabrication of stories

Some teachers felt that some students had not put enough effort into their essays, their written work being poorly presented and incomplete. A few teachers expressed difficulty in dealing with student stories that might have been fabricated. Annoyance with typing errors and suspected fabrication of stories is extremely important as it may impact on the facilitator’s perception of his student’s honesty and diligence. In turn this may affect the facilitator’s interpretation of the student’s reflection and limit the depth of enquiry around his reported experiences.

*“Some students weren’t motivated by this activity. They didn’t put enough effort into completing this so their work was less valuable for them.” (T23)*

*“The writing might not be about their real stories or feelings. (Perhaps) they just tried to make up a nice story. I felt that some students wouldn’t want to show anything that was negative or wouldn’t complete the essay honestly, so they might write fabricated stories. I felt that facilitating those students was very difficult.” (T10)*

However, some teachers felt that incomplete work was a consequence of the fact that most Thai students are unfamiliar with writing reflective essays.

*“Many Thai students are not trained to express their ideas and feelings freely, so it is difficult for them to show their thoughts and feelings through reflective essays.”(T8)*

### Perceived benefits toward students

Most teachers remarked that this activity was interesting as well as being valuable and appropriate for students in the following two areas.

#### Promotion of learning from individual significant experiences

Two aspects of this activity were cited by a teacher as being interesting because they differed from traditional learning methods: 1) Learning was based on the unique experience of each student and guidance was individualized or tailored to this. 2) Learning experiences included not only biomedical content but also focused on other areas of medical education such as professionalism, ethics, and communication skills. This reinforces students’ attitudes in these areas.

Students had protected time to think about their experiences to obtain a deeper understanding of themselves and their interaction with their environment.

*“One student said that if he hadn’t done this activity he wouldn’t have had an opportunity to think about this experience or explore within himself what had happened during the past year. Finally he was able to actually realise how and why he feels proud that patients he has taken care of have become better.” (T12)*

Students can organize thoughts and formulate systematic personal ideas that differ from grumbling about bad events.

*“….They are able to get new and appropriate perspectives that come from gathering and analyzing scattered ideas and thoughts.” (T10)*

This activity provided students with an opportunity to validate and manage their emotions.

*“Some students examined the guilt and regrets that occur in this year in order to successfully resolve their past conflicts and to feel better. The reflective process helps them to see what happened to them more clearly. It made them see their guilt and anxiety clearer. Then these feelings faded, as they made themselves clear. One example was a student who wrote about her first patient. She met and talked to him just for a few days, then the patient died. He died during the night shift. In the morning she came to see him but he wasn’t there anymore. I think writing something like this had helped her. She realized that she felt sorry but then she gradually understood the process that will go on. She began to adjust herself slowly and got over it eventually. I don’t know, when we sit down and write, we have to conceptualize and think back and forth to a deeper level I would say. She revisited her feeling towards past situations and her thoughts at that time, then compared these with the present.” (T17)*

#### Expanding collaborative learning

Unlike other teaching encounters, during small group discussion teachers understood their students better and felt connected with them.

*“It is good to share reflective essays among students. We can understand the student’s real self. This is a channel to connect with some students who are not good at verbal expression.” (T10)*

The teachers could use this opportunity to give specific guidance to students.

*“Through this ‘compulsory’ activity, students and teachers have a formal channel for interactive communication. There are no other formal channels that allow students to talk with us about their first clinical year experiences. The students are able to express their perceptions and feelings. This will help teachers understand and give them specific advice.” (T6)*

In the group process, students not only questioned their own decisions but gauged what was right from other people’s perspectives. Deeper interpersonal connection between students also occurred in the group process. As well as providing a channel for students to share their experiences during the transition year to clinical clerkship, the activity also provided an opportunity for students to understand and support each other in the group process.

*“A student with a reputation for being flippant surprised his classmates with his deep thoughts and feelings. Now his friends can know him better.” (T24)*

### Impact on the personal and professional development of instructors

Teachers recognized that this activity was valuable for students but most of them also felt that it had an impact on their own personal and professional lives. Some commented that the experience of facilitating reflective learning had led to changes in the ways they viewed their teaching styles and fostered a renewed interest in using this technique in their daily lives.

#### Gaining confidence to use this strategy in their teaching

Using reflection in teaching was not viewed as something new by some teachers and joining this activity affirmed or reinforced their view of its value in the undergraduate medical curriculum.

*“I think there should be fewer lecture sessions in our curriculum. The students should learn from their experiences and reflect on these. The deeper learning promoted by this activity results in more change, better understanding and long term retention of ideas.” (T4)*

*“Medicine is not only a science but also an art. Using reflection in medicine is a bridge for the gap between these two. Sometimes students have to face a situation that is not straightforward. They have to use their ‘heart’ to understand and cope with this. This technique will help students to deal with individual difficult experiences.” (T8)*

Most of them found that the younger Thai medical students were able to use reflection much better than they had expected. They recognised how to use reflection in innovative ways and felt more confident about making more use of this method in their teaching sessions.

*“Reflection should be added to the geriatric rotation. Students who care for older patients with chronic diseases should have an opportunity to reflect on their experiences. Or on home visits, if students come across any problem they should have a place to express their feelings or speak out about the problem. Finally they can view the problem systematically.” (T23)*

#### Organizational change

Most instructors did not use reflection in other aspects of their work. Joining this activity stimulated one instructor to implement more reflective activities within his department as a routine practice.

*“I would like to do it (reflection) more. It should be incorporated in most activities. Everyone should respect and be aware of different perspectives within our organisation.” (T6)*

## Discussion

This study aimed to explore medical teachers’ views on the introduction to an undergraduate medical curriculum of an exercise in which students write reflectively about significant events. Although this activity received high student satisfaction, introducing the exercise as a regular feature of our curriculum would impact on teachers due to the need for new skills and demands on their time. In the early phase of implementing this activity teachers expressed concerns about their ability and confidence as facilitators, their ability to deal with emotions raised within their groups, the effectiveness of the activity, and poor presentation of student work.

Most teachers, however, felt that the new activity was beneficial to students by giving them a deeper understanding of their significant events and contributing to their development of a professional identity. It was also felt valuable for students to have their feelings, thoughts, and behaviour validated as part of a group learning process. This is consistent with results of previous studies addressing benefits to students [4, 5, 19].

In addition, we found interesting benefits of this activity for the teachers. They valued the new approach as a tool for their own professional and personal development and were eager to use reflection both in other teaching situations and in their personal lives. The facilitation of student reflection led teachers in turn to reflect on their own roles. For example, being facilitators rather than transmitters of information, detecting student problems that required further attention and being good role models for students. We also identified experiences perceived by the teachers as rewarding. For example, teachers were touched by their students’ stories and felt professionally recharged.

Insights gained from this study are relevant to faculty development and have influenced the training and support we give to our teachers. This is congruent with a study of reflective sessions in an undergraduate nursing curriculum which addressed a lack of appropriate skills for being a preceptor [23]. Many teachers in our study raised concerns over the skills needed for facilitating reflection, especially managing the tension between preserving confidentiality and maintaining engagement, developing a group dynamic that encourages students to participate, and establishing a balanced role between facilitation and teaching. An appropriate faculty development programme in these areas that allows teachers to explore these concerns and to develop coping strategies should also help them to feel comfortable with a more student-centred approach.

Several teachers were concerned that students had fabricated their stories. This was in accord with findings from Pee’s study [19] in which students and dentist tutors expressed doubts that students would complete reflection honestly. For example, students might try to avoid discussion of upsetting incidents or events that might reflect negatively on them. Teachers should be aware that this could arise from a lack of student understanding of the objectives of the activity, an inability to identify significant problems, or anxiety about achieving a particular grade. A faculty development programme would have made them capable of exploring these issues rather than simply pointing out possible misdeeds. It is the teachers’ role to discover and solve these problems.

A previous systematic review addressed the limited outcome from implementing reflective activity in undergraduate medical curricula [24]. However, the bias and confounding factors in education research make it difficult to establish whether or not reflection enhances long term competence through change in clinical practice. Few teachers commented about the effectiveness of using this strategy, but some expected changes in medical student thinking or behaviour as a consequence of this exercise. Further research in our setting should, therefore, address the effects of this activity on short term student outcomes, such as self-awareness or professional thinking.

### Implications

Several implications arise from our study. Both teachers and students viewed the reflective activity as worthwhile, justifying its addition to other courses in our setting. Most of our teachers did not have experience of reflective learning when they were medical students. They were, therefore, unfamiliar with facilitating reflection and were unaccustomed to dealing with the powerful emotions that can arise. This warrants a comprehensive faculty development programme to equip them with appropriate techniques and tools, including the raising of teacher awareness of group dynamics and individual student behavior together with ways of managing these appropriately. Finally, teachers should be given ongoing support, including the provision of feedback on individual performance.

### Strengths and limitations

We used a qualitative method to collect and collate the perceptions and experiences of teachers involved in introducing, implementing and facilitating a reflective writing activity as part of a new curriculum. To the best of our knowledge, this is the first research in medical education to comprehensively address these issues.

Our study has some important limitations. Firstly, as all participants were from a Family Medicine Department, their perspectives might not be generalized for faculties in other fields and other settings. Due to these limitations, further study should assess whether the perceived benefits and concerns we discovered would be similar among teachers in different settings and fields of practice. Dissemination of our findings within the Faculty of Medicine, Ramathibodi Hospital has resulted in other departments showing an interest in formally adopting our strategy. Workshops have been run to introduce facilitation of reflection to teachers in other fields but the impact of this has yet to be assessed. Secondly, all teachers participating in this study were Thai and their expression of feelings may differ from teachers in other cultures. Lastly, the potential for interviewer bias might have influenced the views of participants during the interviews. We attempted to reduce this possibility by training the interviewers, by limiting their dialogue to questions and clarifications, and by instructing them to avoid expressing opinions.

## Conclusions

Medical teachers perceived benefits both to students and themselves from reflective writing with small group discussion. Before implementing this approach in other courses, a comprehensive faculty development programme should be commenced in order to improve the ability and confidence of teachers as facilitators.

### Ethics approval and consent to participate

Permission to conduct this study was obtained from the Institutional Review Board of Faculty of Medicine, Ramathibodi Hospital, Mahidol University.

### Availability of data and materials

Data will not be shared due to restrictions stipulated by the ethics committee when approving the study.
